# Intact Visual Correlation Detection in Autistic Adults

**DOI:** 10.1002/aur.70339

**Published:** 2026-08-03

**Authors:** Lukas Vogelsang, Marin Vogelsang, Cindy E. Li, Margaret Kjelgaard, Annie Cardinaux, Charles A. Nelson, Pawan Sinha

**Affiliations:** ^1^ Department of Brain and Cognitive Sciences Massachusetts Institute of Technology Cambridge Massachusetts USA; ^2^ Hock E. Tan and K. Lisa Yang Center for Autism Research at Massachusetts Institute of Technology Cambridge Massachusetts USA; ^3^ Department of Communication Sciences and Disorders Bridgewater State University Bridgewater Massachusetts USA; ^4^ McGovern Institute for Brain Research Massachusetts Institute of Technology Cambridge Massachusetts USA; ^5^ Department of Pediatrics Harvard Medical School Boston Massachusetts USA; ^6^ Division of Developmental Medicine Boston Children's Hospital Boston Massachusetts USA; ^7^ Harvard Graduate School of Education Cambridge Massachusetts USA

**Keywords:** autism spectrum disorder, coincidence detection, sensory correlations, temporal processing, visual perception

## Abstract

The ability to detect recurring temporal co‐occurrence, or temporal correlation, of sensory events is fundamental for organizing perceptual input into coherent representations. Although past work has revealed differences across several aspects of temporal processing in autism, it is unknown whether autistic and non‐autistic individuals differ in the key computation of correlation detection. Here, we examined this question in the visual domain using an online task. A total of 143 age‐ and sex‐matched adults (73 autistic, 70 non‐autistic) viewed sequences in which three visual elements stochastically alternated between bright and dark states. Two of these were disks, and the third was a surrounding annulus. Participants indicated which of the two disks co‐occurred more reliably with the annulus. Accuracy increased systematically with correlation strength in both groups, but there were no significant group differences and no negative association between performance and autistic trait scores when controlling for nonverbal IQ. These findings suggest that visual correlation detection, under the conditions tested here, is preserved in autistic adults. This points to a key aspect of temporal processing in autism that may provide a stable foundation for perceptual organization even amid challenges reported in other timing‐related domains, such as higher‐level temporal prediction. These results set the stage for future research to identify the mechanisms underlying both preserved and divergent temporal processes, and to translate these findings into evidence‐based support that builds on the observed strengths of autistic individuals.

## 1 Introduction

Imagine participating in a video conference call with several people talking at once. You see their facial movements on the screen and you hear their voices. Even in the absence of binaural sound localization cues (i.e., interaural intensity and time differences), you can tell which voice belongs to which person by comparing the visual and auditory sensory streams over time. This ability to match sensory signals across time enables the binding of what one sees and hears into a coherent representation. The same principle applies even within a single modality; for instance, when two lights repeatedly flash together or when several features of an object change simultaneously. In each case, detecting recurring temporal co‐occurrence of events allows observers to infer that sensory signals originate from a common source, even when different cues might suggest otherwise (Parise et al. 2012, 2013; Parise and Ernst 2016). This illustrates how the detection of recurring temporal co‐occurrences (or “correlations”) over time constitutes a cornerstone of perceptual organization. Without the capacity to accurately detect and exploit temporal correlations in the environment across timescales, an individual would struggle to make sense of a temporally dynamic world. To understand the relevance of this capacity to autism, it is helpful to first consider the broader landscape of temporal processing findings reported in the literature.

Given the central role of temporal processing in perceptual organization (Sinha et al. 2025), it is noteworthy that several studies have reported that autistic individuals experience difficulties in certain aspects of this domain. Systematic reviews point to a heterogeneous pattern of findings, indicating that autistic individuals often show typical performance on basic perceptual timing measures but more frequent challenges in multisensory, motor, and higher‐level temporal abilities (Stevenson et al. 2016; Chan et al. 2016; Allman and Meck 2012; Casassus et al. 2019; Allman and Falter 2015). Specifically, findings reported for low‐level perceptual timing tasks, such as unimodal temporal order judgment or brief interval discrimination, are mixed, with many studies in both adults and children reporting no group differences (e.g., Poole et al. 2021; Wallace and Happé 2008; Mostofsky et al. 2000). In contrast, autistic individuals tend to exhibit more differences in audiovisual temporal processing, including wider temporal binding windows, reduced benefits of multisensory integration, and weaker audiovisual speech integration (Feldman et al. 2018; Jertberg et al. 2024; Stevenson et al. 2016; Foss‐Feig et al. 2010), although some of these effects attenuate with age (Ainsworth and Bertone 2023). Difficulties are also evident in higher‐level temporal abilities, including time‐based prospective memory and diachronic thinking—the ability to understand how events unfold over time—in autistic adults (Altgassen et al. 2012; Kretschmer et al. 2014) and children (Boucher et al. 2007). Autistic individuals also show less accurate or more variable performance in sensorimotor synchronization and rhythmic tapping tasks, both in children (Morimoto et al. 2018) and adults (Vishne et al. 2021; Kasten et al. 2023; Cannon et al. 2024). These challenges have been linked to either weaker real‐time correction of sensorimotor errors (Vishne et al. 2021; Kasten et al. 2023) or noisier internal timekeeping (Cannon et al. 2024). Visuomotor tasks with temporally dynamic objects, such as ball catching, have similarly revealed reduced performance (Park et al. 2024; Ament et al. 2015; Whyatt and Craig 2012). Some recent studies have investigated these temporally unfolding processes within the framework of prediction. As comprehensively reviewed by Cannon et al. (2021), many, though not all, studies suggest that autistic and non‐autistic individuals differ in how they form associations between antecedents and consequences and in how they use probabilistic cues to anticipate events. These observations align with previous theoretical proposals that differences in temporal prediction may account for several core characteristics of the autism phenotype (Lawson et al. 2014; Pellicano and Burr 2012; Sinha et al. 2014; Van de Cruys et al. 2014). Importantly, despite the high prevalence of ADHD in autism, attentional variability is not consistently measured or controlled for in temporal‐processing studies, which adds complexity to interpreting some findings in the prediction literature.

Against this broader backdrop, surprisingly little attention in autism research has been given to the foundational computation of temporal correlation detection, which describes the detection of recurring co‐occurrences of sensory signals to determine how strongly they are related (Parise et al. 2012, 2013; Parise and Ernst 2016). This capacity rests on at least two processes: extracting whether sensory events coincide in time and integrating these co‐occurrences across several instances to detect consistent patterns. Like many classic prediction and probabilistic‐learning tasks, correlation detection requires integrating information over time. However, it involves accumulating statistics of *simultaneous* co‐occurrences rather than of lagged contingencies between antecedents and consequences. Moreover, it is retrospective rather than anticipatory, proceeds without feedback during a trial, and imposes no real‐time updating, speeded decision making, or fast motor responses. As such, while correlation detection is itself a particular form of statistical learning in that it requires extracting regularities from information distributed over time, it differs from the forward‐predictive demands that characterize many higher‐order predictive processes, such as anticipating future events and updating expectations across trials using feedback. Indeed, as proposed by the noted neuroscientist Horace Barlow (Burr and Laughlin 2020), the capacity for temporal correlation detection may reflect the cortex's general strategy for identifying “suspicious coincidences” in sensory input to uncover meaningful structure in the environment (Barlow 1994).

If the important operation of temporal correlation detection were found to differ between autistic and non‐autistic adults, it could help explain consequences for various other facets of temporal processing and prediction. Conversely, if correlation detection were found to be preserved, it would imply that differences rather emerge at higher levels of processing, for example when tasks explicitly require the prediction of future events or trial‐based learning with feedback.

Here, we investigated whether the accuracy of temporal correlation detection differs between autistic adults and typically‐developing peers. To this end, we conducted an online study that examined how accurately a cohort of autistic and non‐autistic adults judged which of two flashing disks was more temporally correlated with a surrounding annulus. Specifically, we hypothesized to find reduced overall correlation‐detection performance in the autistic group, as well as more pronounced performance reduction with lower temporal correlation strength. In addition, we examined associations between task performance and self‐report questionnaire measures of autistic traits and prediction‐related experiences.

Throughout the manuscript, we primarily use identity‐first language, such as “autistic” and “non‐autistic,” to reflect the preferences of many autistic individuals (Bottema‐Beutel et al. 2021).

## 2 Methods

### 2.1 Participants

A total of 143 adults (73 autistic and 70 non‐autistic), aged 18–45 years, consented to participate in this online study, completed all study components, and met the inclusion criteria below. All components of the study, including screening questionnaires, behavioral measures, and experimental procedures, were conducted on participants' personal computers.

Participants resided in the United States and reported English as their native language. Exclusion criteria for both groups included any history of seizures, significant head trauma, premature birth, or uncorrected sensory impairments (hearing or vision). Nonverbal intellectual ability was characterized using the “Test My Brain” (TMB) nonverbal matrix reasoning task (Germine et al. 2012; Singh et al. 2021), with a minimum score of 20 required for inclusion (see Section 2.2 for details). TMB scores serve as a working proxy for nonverbal IQ and henceforth are referred to as such.

Participants were excluded from the non‐autistic group if they reported any diagnosis of mood, psychiatric, or anxiety disorders, or for the use of antipsychotic medication. Reporting a diagnosis of attention‐deficit/hyperactivity disorder (ADHD) was an exclusion criterion for the non‐autistic group, but not for the autistic group. As ADHD frequently co‐occurs with autism, autistic participants with ADHD diagnoses were included, and separate analyses examined any potential differences between the ADHD and non‐ADHD autism subgroups.

Independent autistic adults were recruited through the Simons Foundation Powering Autism Research for Knowledge (SPARK) database, a national resource that maintains phenotypic and genetic information on a large cohort of individuals with an autism diagnosis (Feliciano et al. 2018). Eligibility was first determined using SPARK's characterization measures, and those meeting initial criteria were invited via e‐mail to complete a more detailed screening questionnaire specific to this study. A total of 117 SPARK individuals completed the screening questionnaire. Of these, 74 fully eligible autistic participants successfully completed all study components on their computer, and 73 were included in the analyses (one participant was excluded due to not meeting the nonverbal IQ criterion).

Non‐autistic adults were recruited through the online participant recruitment platform Prolific (https://www.prolific.com). A total of 100 individuals completed the screening questionnaire, with 75 fully‐eligible participants completing all parts of the study, and 70 included in the analyses (5 were excluded for not meeting the IQ criterion).

Our recruitment process yielded groups that were closely matched in sample size (73 autistic, 70 non‐autistic), sex distribution (37M/36F vs. 35M/35F), and age (31.5 vs. 31.4 years). However, as further detailed in Sections 2.4 and 3.1, the ASD group had significantly higher IQ scores, which correlated significantly with task performance. We accounted for IQ in two ways: by including it as a fixed effect in an analysis of the full sample (73 autistic and 70 non‐autistic adults) and, separately, by analyzing a subset of participants (40 adults per group) matched precisely on IQ (TMB) and sex. The results of both analyses are comparable. Table 1 summarizes the participant characteristics of the full sample and the subset.

**TABLE 1:** Characterization of the autistic (ASD) and non‐autistic (NA) group, before and after sub‐selecting to achieve an exact IQ and sex match.

	Full sample		IQ‐ and sex‐matched sub‐sample	
	ASD	NA	ASD	NA
Sample size	73	70	40	40
Age	31.5 (SD = 7.0)	31.4 (SD = 7.0)	31.8 (SD = 6.9)	31.8 (SD = 7.12)
Sex assigned at birth	37M/36F	35M/35F	24M/16F	24M/16F
IQ scores	29.5 (SD = 3.5)	26.9 (SD = 3.2)	27.9 (SD = 3.3)	27.9 (SD = 3.3)
Nr of ADHD participants	31 (out of 73)	0 (exclusion criterion)	15 (out of 40)	0 (exclusion criterion)

*Note:* None of the non‐autistic individuals had ADHD, as this was an exclusion criterion for the NA group. The IQ scores differed significantly between groups for the full sample (*p* < 0.001).

### 2.2 Setup of the Online Study

Screening, demographic surveys, and three characterization measures were administered via Qualtrics (qualtrics.com). The first measure used was the Test My Brain (TMB) nonverbal reasoning task (Germine et al. 2012; Singh et al. 2021), which served both as an inclusion criterion (minimum score ≥ 20) and as an index of nonverbal intellectual ability. In addition, participants also completed the Comprehensive Autistic Trait Inventory (CATI; English et al. 2021, 2025), a validated 42‐item self‐report questionnaire measuring autistic traits across the six domains of social interaction, communication, social camouflage, self‐regulation, cognitive flexibility, and sensory sensitivity. Higher CATI scores indicate a higher degree of autistic traits. Finally, participants completed the Prediction‐Related Experiences Questionnaire (PRE‐Q; O'Brien et al. 2025), which assesses everyday prediction‐related experiences in sensory, motor, and social contexts, with higher composite scores indicating higher levels of prediction abilities.

The main experimental task of the current study, described in Section 2.3, was implemented in PsychoPy (Peirce et al. 2019) and hosted via Pavlovia (pavlovia.org). Participants were instructed to complete the session on a personal computer in a quiet, distraction‐free environment. The experimental task was presented in full‐screen mode, and instructions were provided both as written text and as audio playback to facilitate comprehension.

### 2.3 Experimental Paradigm

**FIGURE 1:**
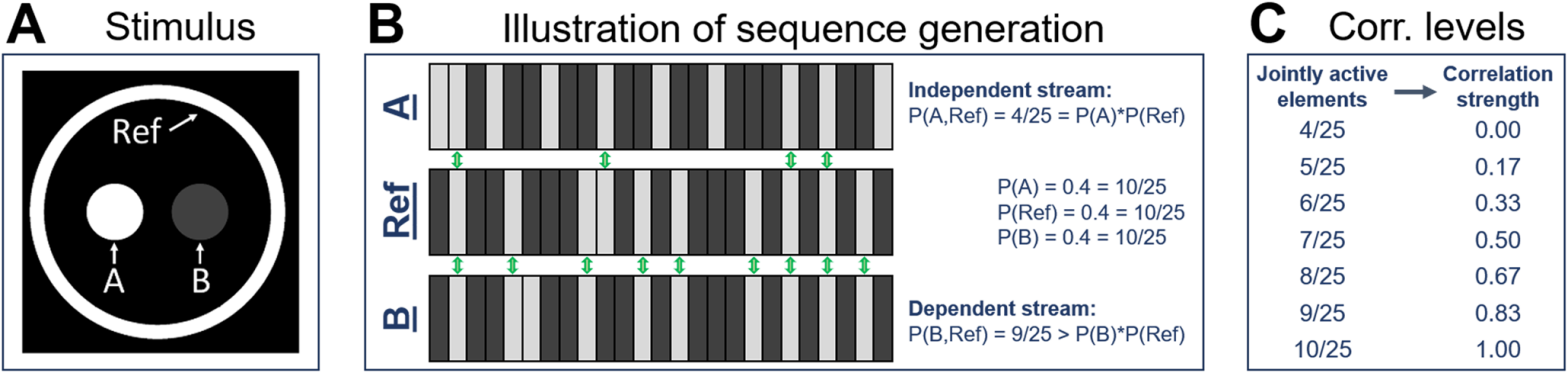
(A) Schematic of the experimental stimulus display. Two disks (A and B) were surrounded by a flashing annulus (Ref). Participants judged which disk's flashing pattern was more correlated with the annulus during a 10‐s sequence (trial). (B) Illustration of sequence generation. All streams had a base rate of *p* = 0.4. In the example shown, disk A was independent of the reference (*p* = 0.16; corresponding to 4 jointly active elements—indicated by green bars between A and Ref), whereas disk B shared one of six levels of dependency with the reference (*p* = 0.20–0.40; corresponding to 5–10 jointly active elements depending on the sequence; in this example, there were 9). (C) Matching between jointly active elements to correlation levels, from full independence (4/25 = 0.16; corresponding to what we refer to as a correlation level of 0) to full dependence (10/25 = 0.4; corresponding to a correlation level of 1). In our example, stream B and Ref, with 9 jointly active elements, exhibit a strong correlation level of 0.83.

Our experimental paradigm comprised a visual correlation detection task in which participants evaluated the temporal co‐occurrence of two flashing disks and a surrounding annulus (Figure 1A). On each trial, participants viewed a sequence of two candidate disks (A and B), both positioned within a larger reference annulus (Ref) on a dark‐gray background. The luminance of all three elements alternated between “active” (bright) and “inactive” (dark) states, according to predetermined binary sequences. After each sequence (or trial), participants indicated which disk (A or B) was flashing more congruently with the annulus.

Each trial contained 25 sequence elements, and each element was presented for 0.4 s. During an inactive state, the element remained dark for the full 0.4 s. During an active state, it was presented bright for the first 0.2 s. Thus, one trial lasted 10 s (25 sequence elements × 0.4 s). All three streams had the same base probability of being active (P(A) = P(B) = P(Ref) = 0.4), corresponding to 10 out of 25 “active” elements.

As illustrated in Figure 1B, one stream (in the illustration, A) was generated to be entirely independent of the reference stream, such that their joint probability of being active together equaled the product of their base probabilities (P(A, Ref) = 0.4 × 0.4 = 0.16; corresponding to 4 out of 25 jointly active elements in the sequences). The other stream (in the illustration, disk B) was constructed to co‐occur with the reference stream more often than expected by chance (i.e., with joint probabilities > 0.16). Six joint probabilities were tested in our experiment: 0.20, 0.24, 0.28, 0.32, 0.36, and 0.40 (corresponding to 5–10 out of 25 jointly active elements in the sequences). As a joint probability of 0.16 corresponds to a correlation no more than chance, we refer to it as a correlation level of 0. Moreover, as a joint probability of 0.4 corresponds to a correlation level of 1 (fully identical sequences), the six joint probabilities map onto correlation levels 0.17, 0.33, 0.50, 0.67, 0.83, and 1.00 (Figure 1C).

For each level, six unique sequences were created, yielding a total of 36 trials. Sequences were pre‐generated and identical across participants, but their order of presentation was randomized for each participant.

Prior to the actual task, participants were presented with instructions and then completed a set of practice trials with highly correlated sequences to ensure task comprehension. Participants were permitted to pause between trials if needed, for as long as they desired.

PsychoPy allows for high temporal precision (Bridges et al. 2020), and we also verified that the stimuli displayed as intended across devices prior to data collection. In addition, because each element was displayed for a minimum of 200 ms and stimuli were presented as pre‐generated, pre‐loaded full‐screen images rather than rendered in real time, the paradigm does not heavily depend on millisecond‐level display precision. Moreover, the correlation structure that participants judged is defined by which elements are jointly active and should therefore not be affected even in the occasional event of variable frame‐level timing.

### 2.4 Statistical Analysis

In the full sample of 73 autistic and 70 non‐autistic participants, groups were closely matched in sex assigned at birth; henceforth “sex” (ASD: 37 male, 36 female; NA: 35 male, 35 female) and age (ASD: mean 31.5 years; NA: mean 31.4 years). However, as shown in Table 1, the groups differed significantly in IQ, which also correlated significantly with task performance. Consequently, any apparent group differences in the full sample may be driven by underlying differences in IQ. We addressed this in two complementary ways. First, we analyzed the full sample using a linear mixed‐effects model that included nonverbal IQ as an additional fixed effect. Second, we carried out our analysis on an IQ‐ and sex‐matched subset of 40 autistic and 40 non‐autistic participants, created by exact matching of TMB scores and sex on an individual‐by‐individual basis. The results of both analyses were comparable, indicating that they do not depend critically on the specific handling of IQ confounds.

Group comparisons of task performance were conducted using a linear mixed‐effects model, with performance as the dependent variable; group, correlation level, and their interaction as fixed effects; and random intercepts for participants as random effects. A model with random slopes for correlation level was not favored by BIC. For the full‐sample analysis, nonverbal IQ was included as an additional fixed effect.

### 2.5 Ethical Review

The study protocol was approved by the MIT Committee on the Use of Humans as Experimental Subjects (COUHES) and conducted in accordance with the Declaration of Helsinki. Participants could withdraw at any time and were compensated for the portion of the study they completed. Compensation was not contingent on performance. All participants provided informed consent electronically.

## 3 Results

### 3.1 Performance on the Correlation Detection Task

**FIGURE 2:**
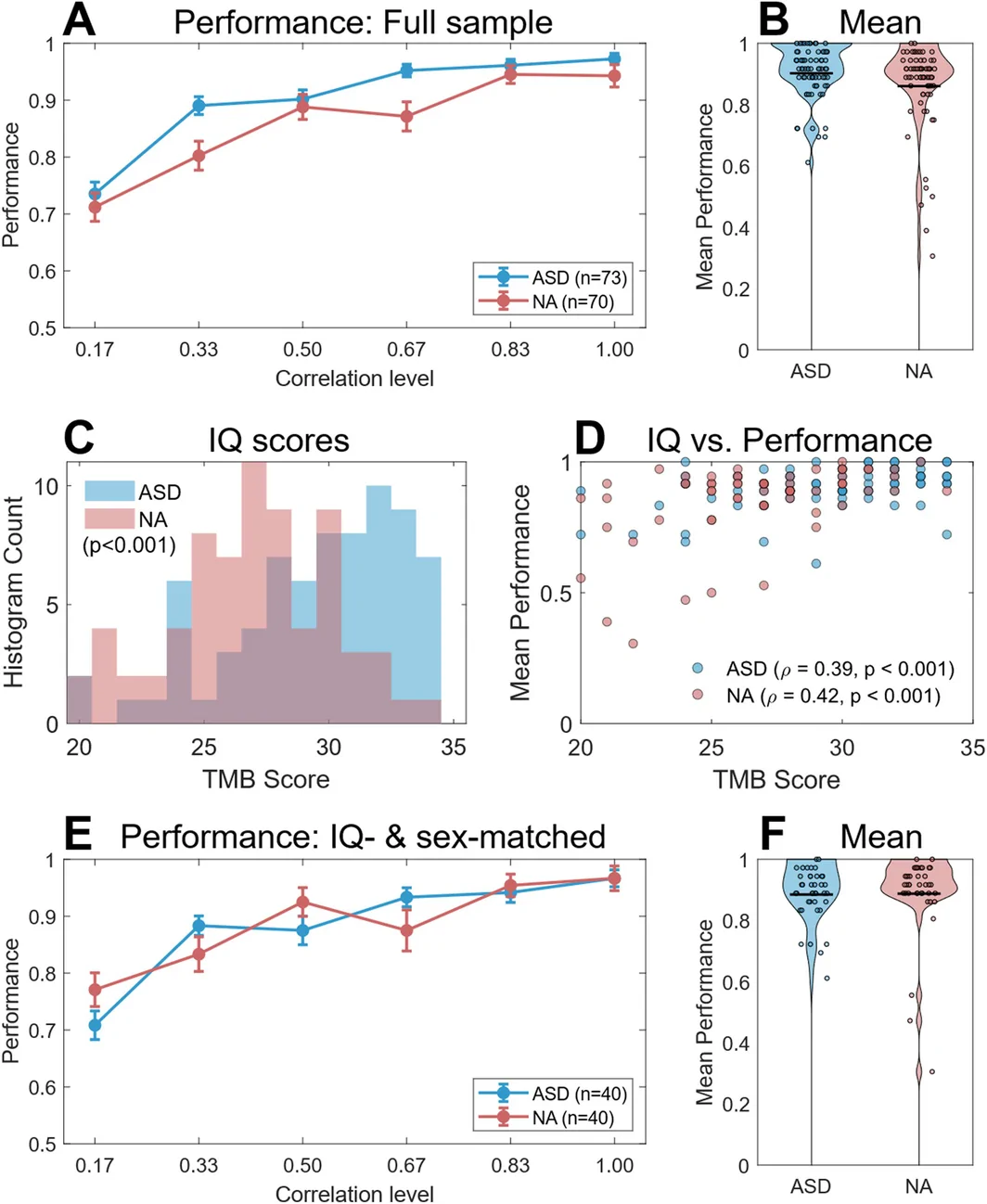
(A) Similar performance (mean ± standard error) as a function of correlation level for the full sample of autistic (ASD, *n* = 73) and non‐autistic (NA, *n* = 70) adults. (B) Full distribution of performance when averaged across correlation levels. (C) Histogram of the full set of 73 ASD and 70 NA adults on IQ (TMB) scores, revealing significantly higher IQ scores for the ASD group. (D) Positive correlation between IQ scores and task performance for both groups. (E, F) Replication of panels A and B with a sub‐selected sample (*n* = 40 per group) that is matched individually on IQ scores and sex. Performance is even more similar after matching.

Figure 2A,B shows performance on the correlation detection task for the full sample of 73 autistic and 70 non‐autistic adults. Contrary to our hypothesis, autistic adults performed similarly to non‐autistic adults; in fact, the ASD group mean accuracy was slightly higher (90.2% in the ASD group vs. 86.0% in the NA group). However, the autistic group had significantly higher nonverbal IQ as indexed by TMB (Figure 2C; Mann–Whitney *U*, *p* < 0.001). This is especially important to note as IQ scores were positively related to task performance for both autistic (*ρ* = 0.39, *p* < 0.001) and non‐autistic (*ρ* = 0.42, p < 0.001) groups (Figure 2D). Thus, IQ differences could potentially drive group differences observed. We addressed this in two ways, to ensure the stability of our main findings.

First, we analyzed the full sample using a linear mixed‐effects model that included nonverbal IQ as a fixed effect alongside group, correlation level, and their interaction. As shown in Table 2, IQ and correlation level were significant positive predictors of performance (*p*'s < 0.001). However, there was no significant group difference (*p* = 0.556) and no group × correlation level interaction (*p* = 0.506).

**TABLE 2:** Estimation of fixed effects from the linear mixed model for the full sample of 73 autistic and 70 non‐autistic participants when IQ is added as a fixed effect.

Term	Estimate	SE	df	*t*	*p*
Intercept	0.312	0.074	144.0	4.219	< 0.001
IQ	0.015	0.003	144.0	5.743	< 0.001
Group	0.008	0.013	434.4	0.590	0.556
Correlation_level	0.259	0.015	713.0	17.160	< 0.001
Group × correlation_level	−0.010	0.015	713.0	−0.665	0.506

Similar results emerge from our second analysis, in which we matched a subset of participants exactly for TMB scores and sex on an individual‐by‐individual basis, yielding a sample size of 40 participants (16 females) per group. In this controlled sub‐sample, mean accuracy was even more similar than in the full sample (88.5% in the ASD group vs. 88.7% in the NA group) (Figure 2E,F). Consistent with results of our first analysis, a linear mixed‐effects analysis in this subset revealed a significant effect of correlation level (*p* < 0.001) but no significant group difference (*p* = 0.438) and no group × correlation level interaction (*p* = 0.280) (see Table 3 for details). Thus, across both analyses, the small advantage apparent for the ASD group in the full sample is explained by group differences in IQ scores. When it is accounted for, the groups perform indistinguishably across correlation levels.

**TABLE 3:** Estimation of fixed effects from the linear mixed model for the sub‐sample of 40 autistic and 40 non‐autistic participants.

Term	Estimate	SE	df	*t*	*p*
Intercept	0.745	0.017	205.7	44.384	< 0.001
Group	−0.013	0.017	205.7	−0.778	0.438
Correlation_level	0.241	0.018	398.0	13.068	< 0.001
Group × correlation_level	0.020	0.018	398.0	1.083	0.280

Finally, we addressed the asymmetry in ADHD diagnoses between groups, since ADHD was present in the autistic group but excluded by design from the non‐autistic group. Two analyses suggest the lack of an effect of ADHD. First, within the autistic group, mean performance did not differ between participants with and without ADHD (Supplementary Figure S1; Mann–Whitney *U*, *p* = 0.871 for the full sample and *p* = 0.415 for the sub‐sample). Second, to remove the ADHD variable from the between‐group comparison entirely, we repeated our main analysis comparing the 70 non‐autistic participants with the 42 autistic participants without ADHD, again including IQ as a fixed effect. As shown in Table 4, the results were unchanged: no significant group difference (*p* = 0.587) and no group × correlation level interaction (*p* = 0.359) were observed, while IQ and correlation level remained significant predictors (*p*'s < 0.001).

**TABLE 4:** Estimation of fixed effects from the linear mixed model for the full sample of 70 non‐autistic participants and the non‐ADHD subset of 42 autistic participants when IQ is added as a fixed effect.

Term	Estimate	SE	df	*t*	*p*
Intercept	0.217	0.092	111.9	2.351	0.020
IQ	0.018	0.003	109.0	5.645	< 0.001
Group	0.008	0.015	332.2	0.543	0.587
Correlation_level	0.252	0.018	558.0	13.935	< 0.001
Group × correlation_level	−0.017	0.018	558.0	−0.918	0.359

### 3.2 Relation Between Task Performance and Characterization Measures

**FIGURE 3:**
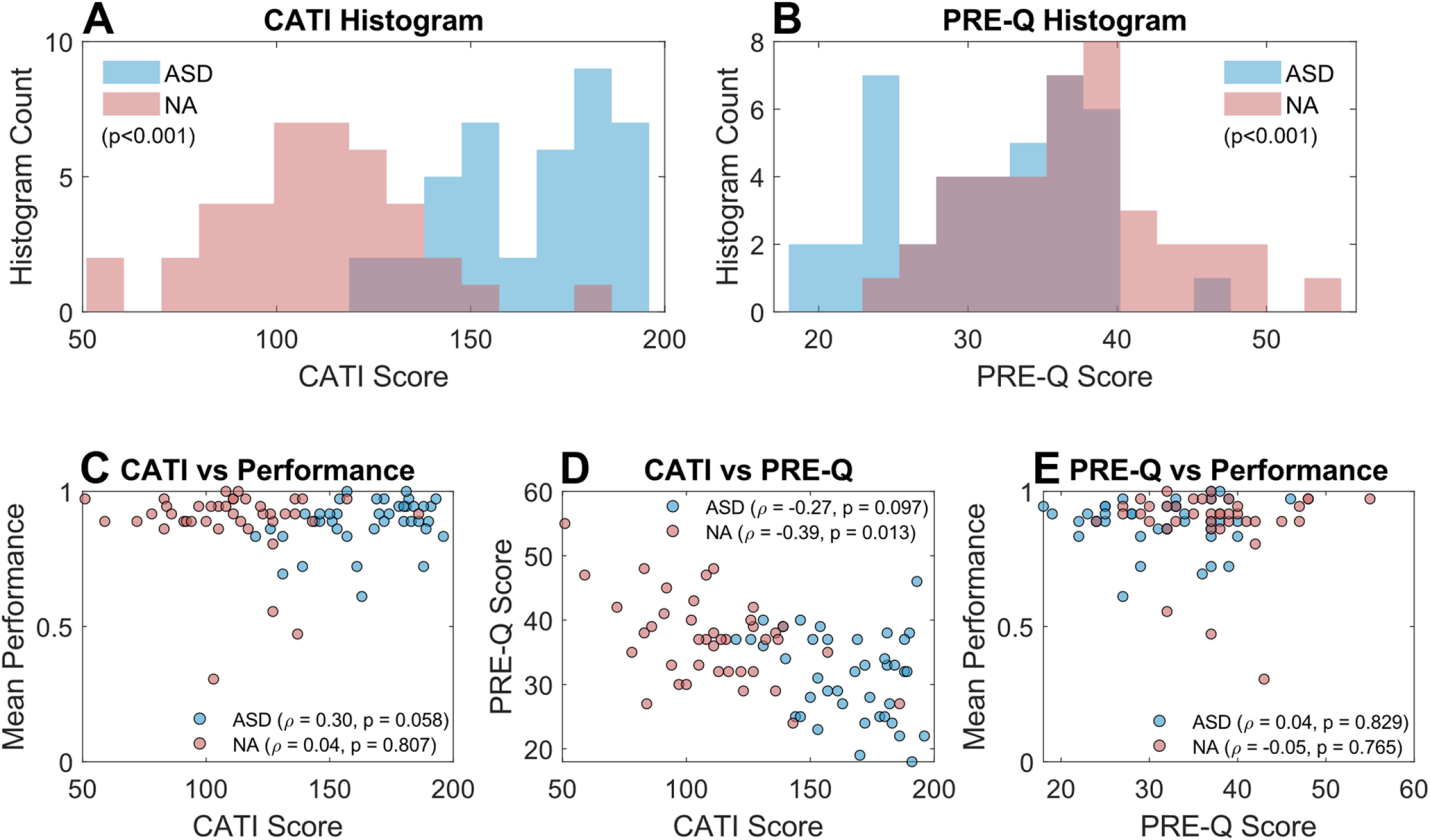
(A, B) Histogram showing distribution of Comprehensive Autistic Trait Inventory (CATI) scores (A) and Prediction‐Related Experiences Questionnaire (PRE‐Q) scores (B) for the IQ‐ and sex‐matched sub‐sample (40 ASD and 40 NA participants). Across both measures, groups differ significantly. (C–E) Scatter plots depicting the within‐group statistical correlation between CATI score and mean task performance (C), CATI score and PRE‐Q score (D), and PRE‐Q score and mean task performance (E).

Within the IQ‐ and sex‐matched sample, we also examined how task performance related to two key characterization questionnaire measures. First, Comprehensive Autistic Trait Inventory (CATI) scores, where higher scores indicate a higher degree of autistic traits, were, as expected, higher in the ASD than in the NA group (Mann–Whitney *U*, *p* < 0.001) (Figure 3A). Notably, and contrary to our hypothesis, CATI scores did not show a negative association with task performance (Figure 3C).

Second, Prediction‐Related Experiences Questionnaire (PRE‐Q) scores, for which lower scores indicate a higher level of prediction‐related challenges, were also significantly lower in the autistic group (Figure 3B; Mann–Whitney *U*, *p* < 0.001). This is consistent with past theoretical accounts emphasizing predictive differences in autism, as well as a prior empirical study of autistic and nonautistic adults who completed the PRE‐Q (O'Brien et al. 2025). Furthering this linkage, as shown in Figure 3D, CATI and PRE‐Q scores are negatively correlated, reaching significance for the non‐autistic group in the sub‐sample of 40 participants (*ρ* = −0.39, *p* = 0.013) but not in the full sample (*ρ* = −0.18, *p* = 0.132), whereas for the autistic group, significance is reached only in our full sample (*ρ* = −0.37, *p* = 0.001). Notably, PRE‐Q scores were not significantly linked to task performance (Figure 3E).

In summary, while group differences are evident on both questionnaires, higher CATI or lower PRE‐Q scores are not systematically linked to lower performance in this task. Qualitatively similar patterns are observed in our full sample of 73 autistic and 70 non‐autistic adults (Figure S2).

## 4 Discussion

In this study, we examined whether autistic adults differ from non‐autistic adults in detecting temporal correlations between sequences of probabilistically co‐occurring visual events. In a sample of carefully matched autistic and non‐autistic adults, we found no evidence for such a difference. As expected, accuracy increased with correlation strength in both groups. A slight numerical performance advantage for autistic adults in the full sample (4.2%) further decreased when controlling for nonverbal reasoning ability (IQ) and sex, and was statistically indistinguishable between groups. Contrary to our hypothesis, the autistic group always performed at least as well as the non‐autistic group.

Together, these findings provide evidence that visual correlation detection, under the paradigm, conditions, and difficulty levels tested here, is preserved in autism. Identifying preserved functions is as important as finding differences, as it helps identify the mechanisms underlying perceptual and predictive function in autism. As Barlow (1994) noted, the cortex's ability to detect “suspicious coincidences” in sensory input is fundamental for detecting structure in the environment. The finding that this capacity is intact in autism is therefore especially noteworthy. It may provide a foundation for perceptual organization and, by implication, help pinpoint where in the perceptual processing hierarchy group differences are more likely to emerge.

In this context, our findings add nuance to the broader literature on temporal processing in autism. Prior reviews have reported that timing is not uniformly affected. Differences are most consistently reported in higher‐order domains, whereas evidence for lower‐level perceptual processing differences is mixed (Casassus et al. 2019). For instance, tasks requiring detection of the non‐simultaneity or temporal order of briefly separated sensory events yield inconsistent results. Some studies report typical performance in autistic participants (de Boer‐Schellekens, Keetels, et al. 2013), others find reduced (Puts et al. 2014; de Boer‐Schellekens, Eussen, and Vroomen 2013) or enhanced performance (Falter et al. 2012), and still others show typical performance in one but impaired performance in another domain (Kwakye et al. 2011). In addition, correlation detection requires not only the detection of simultaneity but depends critically on integrating the statistics of simultaneous occurrences *over time*, especially in sequences with low correlation strength. Our results indicate that this integrative mechanism appears to function typically in our sample of independently living, cognitively able autistic adults over the time scale measured in this experiment and in the visual modality. As stated at the outset, the need for statistical integration of information over time is shared between correlation detection and many classic predictive or probabilistic learning tasks, suggesting that temporal integration of probabilistic events per se is unlikely the locus at which group differences emerge.

Our paradigm deliberately did not place strong demands on classic forward prediction. Specifically, participants did not need to anticipate future events, make timing‐based motor responses, or use feedback to update expectations across trials. Moreover, while many prediction tasks rely on a learned relationship in which an earlier event signals a later one, our task included no such antecedent‐consequence structure. Instead, participants judged how reliably two events co‐occurred in time, rather than how an earlier event predicted a later one. This allowed us to focus on correlation detection as the integration of simultaneous event statistics (of varying probabilities) over time.

Autistic participants in our sample differed markedly in their responses to the Prediction‐Related Experiences Questionnaire, replicating findings from a prior empirical study from our group (O'Brien et al. 2025). This is consistent with broader theoretical accounts hypothesizing predictive differences in autism (Lawson et al. 2014; Pellicano and Burr 2012; Sinha et al. 2014). However, PRE‐Q scores in the present study were not correlated with correlation detection task performance. This lack of association suggests that self‐reported predictive experiences differ substantially from the demands of the present correlation detection task. This does not, however, establish that the correlation detection task is free of predictive components. Instead, a null association may be expected given that the two measures differ in several additional ways. Specifically, the PRE‐Q is subjective, ecological, and concerned with processes that may unfold over extended timescales, whereas our task is objective, psychophysical, and confined to relatively short trial sequences.

Our findings fit within a broader pattern in which autistic individuals often show intact or even enhanced performance in perceptual tasks, such as embedded figure processing, visual search, or pitch discrimination (Shah and Frith 1983; Plaisted et al. 1998; Bonnel et al. 2003; Hisaizumi and Tantam 2024; Hadad and Yashar 2022). They also complement work showing preserved, and in some cases enhanced, implicit statistical learning in autism. For example, autistic adults have been shown to exhibit superior learning of spatial co‐occurrence structure compared to non‐autistic adults (Roser et al. 2015) as well as intact acquisition of sequential regularities in a serial reaction time task (Treves et al. 2024). Even though these paradigms, unlike ours, require learning across, rather than within, trials, they similarly point to preserved mechanisms for extracting structure from the environment. Together, these results indicate that several key processes for detecting regularities in the environment are preserved in autism. By contrast, tasks that more consistently reveal group differences, such as rhythmic tapping or intercepting moving objects, place stronger demands on forward prediction.

It is important to note several caveats, which in turn point to interesting directions for future experimental work. The first concerns the level of difficulty tested. In the present study, we varied correlation level, and thus difficulty, parametrically, and included a maximally challenging condition for the present design (in which the correlated stream had only one more jointly active element with the reference stream than the fully uncorrelated stream), at which performance was between 70% and 80% for both groups and therefore non‐saturated. It is important to note that we did not find a significant interaction effect between group and difficulty level. However, we cannot, on this basis, exclude the possibility that group differences might emerge under more challenging conditions. Accordingly, our conclusion that correlation detection is intact should be understood as being specific to the conditions and difficulty range tested here. Probing finer sensitivity, for instance by introducing more than two candidate streams, lengthening the sequences, or shortening element presentation times, would be a valuable way forward.

Complementing performance‐based analyses, future work could also compare how much of the stimulus sequence needs to be observed before reaching a reliable decision, and whether this differs between autistic and non‐autistic groups even when full‐sequence performance may not. We implemented a related approach in a recent study examining correlation detection in individuals who gained sight late in life, compared to normally sighted controls. There, participants reported a decision during sequence presentation as soon as they felt confident as well as again at its conclusion, allowing first‐decision points to be analyzed in addition to full‐sequence performance (Gupta et al. 2026). In the present study, we used only a single post‐sequence response to avoid introducing potential group differences due to differences in attentional demands of a dual‐response procedure. However, in the future, comparing autistic and non‐autistic individuals on sequences deliberately truncated at varying points would offer a more direct way to test underlying decision‐making dynamics, while also naturally probing more challenging conditions.

Our participant sample also merits further discussion. A notable strength is the approximately equal proportion of females in the sample, which is uncommon in autism research (D'Mello et al. 2022) and enhances the generalizability of our findings. Additional strengths include the careful matching of participants on age, sex, and nonverbal IQ, and the use of strict inclusion criteria that minimize potential confounds. At the same time, the composition of our sample imposes certain limits on generalizability. Autistic participants were independently living, cognitively able adults recruited through SPARK. As such, this group may differ in important ways from autistic individuals who are less likely to participate in online studies, such as those with lower language ability or nonverbal IQ. Consequently, our findings may not fully represent the broader autism spectrum, particularly individuals with more profound support needs and across the full spectrum of language abilities. Future work should test whether similar patterns of preserved temporal correlation detection extend across the full range of autistic experiences and abilities. In addition, examining individuals with other neurodevelopmental conditions would help probe whether the pattern observed here is specific to autism.

Future research should also test whether the findings obtained here generalize across sensory modalities. Temporal processing differences in autism may be more prominent in auditory and cross‐modal contexts (Stevenson et al. 2016). Multisensory correlation detection tasks (Parise et al. 2012, 2013; Parise and Ernst 2016) could reveal whether the preservation observed here extends beyond vision. Additional insight could be gained from developmental and neural investigations. Longitudinal designs could determine whether correlation detection is intact from childhood or whether potential group differences early in life attenuate with age. Finally, neurophysiological methods, such as EEG, MEG, or fMRI, could establish whether autistic and non‐autistic adults show comparable neural mechanisms, such as recruited brain regions, activation strength, and timing, even as behavioral responses appear similar.

Notwithstanding these open questions, the present findings demonstrate that autistic adults detect temporal correlations in visual streams over time as proficiently as non‐autistic adults. These findings point to the value of distinguishing between different aspects of temporal processing. While autistic and non‐autistic adults may differ on certain higher‐level functions, our results indicate that the ability to accurately detect co‐occurring visual events is preserved. This ability may thus support perceptual organization, with group differences arising instead in tasks that place heavier demands on generating or updating forward predictions. Examining the mechanisms underlying both preserved and divergent temporal processes is a fruitful direction for future work. From an applied perspective, the finding of intact perceptual function could also inform evidence‐based support that builds on the observed strengths of autistic individuals.

## Funding

Lukas Vogelsang is supported by a grant from the Simons Foundation International to the Simons Center for the Social Brain at MIT. Marin Vogelsang is supported by the Japan Society for the Promotion of Science (JSPS) Overseas Research Fellowship and the Yamada Science Foundation. Cindy Li and Annie Cardinaux are supported by the Hock E. Tan and K. Lisa Yang Center for Autism Research. Charles Nelson is supported by the NIH (R01 NS120986‐01; P50 HD073961). Pawan Sinha is supported by NIH grant R01EY020517 and the MIT Schwarzman College of Computing.

## Ethics Statement

The study protocol was approved by the MIT Committee on the Use of Humans as Experimental Subjects and conducted in accordance with the Declaration of Helsinki. All participants provided informed consent electronically.

## Conflicts of Interest

The authors declare no conflicts of interest.

## Supporting information


**Figure S1:** Full distribution of performance when averaged across correlation levels, for the subset of ASD participants with (left) and without (right) ADHD, depicted separately for the full sample of 73 autistic individuals (A) and the subset of 40 individuals matched for IQ and sex with the non‐autistic control group (B). For both, differences were not significant (Mann–Whitney *U*; *p* = 0.871 for full sample, *p* = 0.415 for subsample).
**Figure S2:** (A, B) Histogram showing distribution of CATI scores (A) and PRE‐Q scores (B) for the full sample of 73 ASD and 70 NA participants. (C–E) Scatter plots depicting the correlation between CATI score and mean performance (C), CATI score and PRE‐Q score (D), and PRE‐Q score and mean performance (E).

## Data Availability

The analysis code that supports the findings of this study is available in a public Zenodo repository (https://doi.org/10.5281/zenodo.21617657). The participant data are available upon reasonable request from the corresponding author and are not publicly available due to privacy restrictions.
