# Supplementary material for: Intact Visual Correlation Detection in Autistic Adults
Source: Autism Res. 2026 Aug 3;19(9):e70339. doi: 10.1002/aur.70339 (PMC13599692; doi:10.1002/aur.70339)
Supplement: Supplementary file 1 [file AUR-19-0-s001.docx]

**Supplementary material accompanying the manuscript
‘Intact visual correlation detection in autistic adults’**

**Content:**
Supplementary Figures 1-2.

***Supplementary Figure 1.*** *Full distribution of performance when averaged across correlation levels, for the subset of ASD participants with (left) and without (right) ADHD, depicted separately for the full sample of 73 autistic individuals (A) and the subset of 40 individuals matched for IQ and sex with the non-autistic control group (B). For both, differences were not significant (Mann-Whitney U; p = 0.871 for full sample, p = 0.415 for subsample).*

***Supplementary Figure 2. A-B.*** *Histogram showing distribution of CATI scores (A) and PRE-Q scores (B) for the full sample of 73 ASD and 70 NA participants.* ***C-E.*** *Scatter plots depicting the correlation between CATI score and mean performance (C), CATI score and PRE-Q score (D), and PRE-Q score and mean performance (E).*
